# Prognosis of Early-Stage Hepatocellular Carcinoma: The Clinical Implications of Substages of Barcelona Clinic Liver Cancer System Based on a Cohort of 1265 Patients

**DOI:** 10.1097/MD.0000000000001929

**Published:** 2015-10-30

**Authors:** Wei-Yu Kao, Yee Chao, Chun-Chao Chang, Chung-Pin Li, Chien-Wei Su, Teh-Ia Huo, Yi-Hsiang Huang, Yu-Jia Chang, Han-Chieh Lin, Jaw-Ching Wu

**Affiliations:** From the Division of Gastroenterology and Hepatology, Department of Medicine, Taipei Veterans General Hospital, Taipei, Taiwan (W-YK, C-PL, C-WS, T-IH, Y-HH, H-CL); Faculty of Medicine, School of Medicine, National Yang-Ming University, Taipei, Taiwan (W-YK, YC, C-PL, C-WS, H-CL); Division of Gastroenterology and Hepatology, Department of Internal Medicine, Taipei Medical University Hospital, 252, WuHsing St., Taipei 11031, Taiwan (W-YK, C-CC); Division of Gastroenterology and Hepatology ,Department of Internal Medicine, School of Medicine, College of Medicine, Taipei Medical University, 250, WuHsing St., Taipei 11031, Taiwan (W-YK, C-CC); Graduate Institute of Clinical Medicine, College of Medicine, Taipei Medical University, Taipei, Taiwan (W-YK, Y-JC); Division of Chemo-radiotherapy, Department of Oncology Medicine, Taipei Veterans General Hospital, Taipei, Taiwan; Institute of Pharmacology (YC); Institute of Pharmacology, School of Medicine, National Yang-Ming University, Taipei, Taiwan (T-IH); Institute of Clinical Medicine, School of Medicine, National Yang-Ming University, Taipei, Taiwan (Y-HH, J-CW); Division of General Surgery, Department of Surgery, Taipei Medical University Hospital, Taipei Medical University, Taipei, Taiwan (YJC); Translational Research Laboratory, Cancer Center, Taipei Medical University Hospital, Taipei, Taiwan (YJC); Cancer Center, Taipei Medical University Hospital, Taipei Medical University, Taipei, Taiwan (Y-JC); and Division of Translational Research, Department of Medical Research, Taipei Veterans General Hospital, Taipei, Taiwan (J-CW).

## Abstract

The prognostic implication of Barcelona Clinic Liver Cancer (BCLC) substages in Asian patients with hepatocellular carcinoma (HCC) is still obscure.

The aim to this study is to compare the prognoses among HCC patients in different substages of the BCLC stage 0 and A.

We enrolled 1265 treatment-naive HCC patients with BCLC stage 0 or A from 2007 to 2014. Factors in terms of prognoses were analyzed by multivariate analysis.

There were 184, 446, 271, 92, and 272 patients in substage 0, A1, A2, A3, and A4, respectively. After a median follow-up period of 21.0 months, patients in stages 0 and A1 had comparable prognoses (*P* = 0.136). Both of them had significantly higher overall survival rates than those in stages A2–A4 (all *P* < 0.001). Multivariate analysis revealed that the factors associated with mortality were serum albumin ≤3.5 g/dL (hazard ratio [HR] 1.459), alpha-fetoprotein >20 ng/mL (HR 1.863), tumor size >3 cm (HR 1.542), BCLC stage A2–A4 (HR 1.488), and treatment modality. Surgical resection had the highest overall survival rate followed by radiofrequency ablation (RFA) (HR 1.598), transarterial chemoembolization (HR 2.224), and other treatments (HR 3.707). Verus patients receiving RFA, those who underwent resection had a higher overall survival rate and lower recurrence rate especially in BCLC stages A2–A4.

The BCLC-staging system provided good prognostic stratification for early-stage HCC. Patients with a single tumor >2 cm but without portal hypertension or jaundice had similar prognosis to those in BCLC stage 0. Curative therapies—especially hepatic resection—are suggested in early-stage HCC.

## Abstract 2

Supplemental Digital Content is available in the text

## INTRODUCTION

Hepatocellular carcinoma (HCC) is the third most common cause of cancer-related deaths worldwide.^1,2^ In most Asian and African countries, hepatitis B virus (HBV) infection is the major risk factor for HCC, while hepatitis C virus (HCV) infection is the main etiology of HCC in Japan and the West.^3–5^ In recent decades, the prognosis of patients with HCC has improved because more cases are diagnosed and treated at early stages due to the promotion of HCC surveillance for high-risk groups such as chronic HBV or HCV infection^6,7^ as well as increased application of loco-regional treatments.^8–11^

Important factors that may affect the prognosis of patients with HCC include patient factors (such as age and performance status),^12–14^ tumor burden (the size and number of tumors; vascular invasion),^15^ serum alpha-fetoprotein (AFP) level,^16^ liver functional reserve (eg, Child–Pugh score, portal hypertension [PHT], and platelet count),^17–20^ genetic factors,^21–23^ and treatment modalities.^24,25^ To date, there are more than 10 HCC-staging systems proposed by different research groups.^26–29^ Of these, the Barcelona Clinic Liver Cancer (BCLC) staging classification is the only validated staging system that currently links prognostic classification to appropriate treatment options. With internal and external validations, the BCLC staging system exhibits excellent prognostic stratification and is widely recommended as a treatment allocation guideline.^2^

The BCLC very early stage (stage 0) includes patients with single HCC ≤ 2 cm in a well-compensated liver function without PHT. The BCLC early-stage HCC (BCLC A) classification includes patients in Child–Pugh grade A or B with single HCC or with 2 or 3 nodules below 3 cm. It has been further classified into 4 subgroups (A1–A4) according to the PHT status, jaundice, and tumor numbers.^26^ Briefly, BCLC stage A1 consists of patients with a single tumor without PHT and jaundice. A2 is defined as a single tumor with PHT but no jaundice. BCLC stage A3 consists of single tumor with relevant PHT and increased serum bilirubin levels, and BCLC stage A4 was classified as 2 to 3 tumors smaller than 3 cm regardless of PHT or jaundice. However, there is limited data regarding the prognostic implication of substages in BCLC stage A in Asian patients. The goal of this study is to compare the clinical manifestations, treatment modalities, and outcomes between BCLC stage 0 and A1–A4 HCC patients.

## METHODS

### Patients and Follow-Up

**FIGURE 1:**
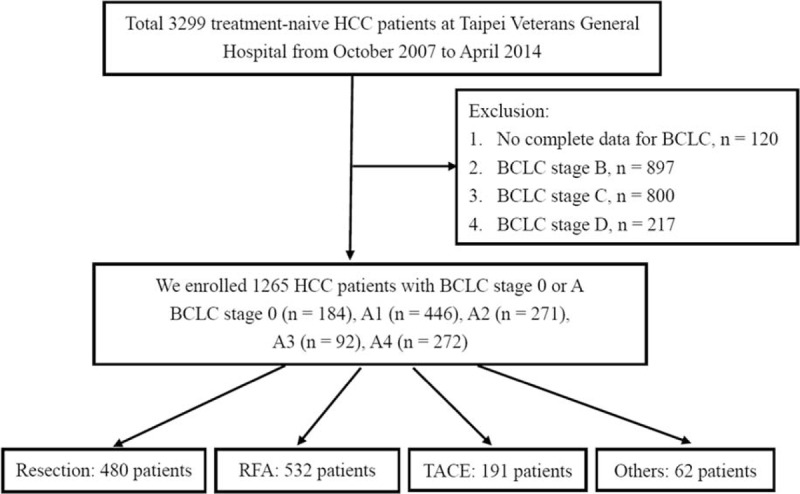
The study flow chart.

This cohort study was prospectively conducted and retrospectively analyzed; it enrolled 3299 consecutive treatment-naive patients who fulfilled the diagnostic criteria of HCC by the American Association for the Study of Liver Disease (AASLD consensus, 2005).^30^ These were enrolled in the cancer registration system at Taipei Veterans General Hospital from October 2007 to April 2014 (Fig. 1). All of the patients were followed up every 3 months until their last visit to the hospital, death or October 31, 2014. Enrolled patients underwent thorough clinical, laboratory, and image assessment. As hepatic venous pressure gradient was not measured in our cohort, PHT was defined according to the criteria proposed by the Barcelona group for patients with the presence of either esophageal varices (EV) detectable by endoscopy and/or splenomegaly (spleen diameter >12 cm by ultrasound) with platelet counts below 100,000/mm^3^.^20,26^ A total of 1265 BCLC stage 0 or A patients were enrolled for the final analysis after excluding patients without complete data for BCLC stage (120 patients) and those in BCLC stage B (897 patients), stage C (800 patients), and stage D (217 patients). Of these, 184 patients had BCLC stage 0, 446 patients had BCLC stage A1, 271 patients had BCLC stage A2, 92 patients had BCLC stage A3, and 272 patients had BCLC stage A4, respectively.

All of the HCC patients were discussed in terms of the diagnosis and treatment strategy at a weekly multidisciplinary meeting. The number of patients undergoing curative treatments (resection surgery and radiofrequency ablation therapy [RFA]) was 480 and 532, respectively. After the physicians explained the advantages, side effects, and prognosis in the various therapy modalities, the number of patients undergoing noncurative treatments of transarterial chemoembolization (TACE) and others (such as best supportive treatment, chemotherapy, sorafenib, radiotherapy, and chemo-radiotherapy) were 191 and 62, respectively. The study complied with the standards of the Declaration of Helsinki and current ethical guidelines. It was approved by the Institutional Review Board of Taipei Veterans General Hospital.

### Biochemical and Serologic Markers

Serum hepatitis B surface antigen and the HCV antibody were tested by radio-immunoassay (Abbott Laboratories, North Chicago, IL) and second-generation enzyme immunoassay (Abbott Laboratories, North Chicago, IL). Serum biochemistries were measured using a Roche/Hitachi Modular Analytics System (Roche Diagnostics GmbH, Mannheim, Germany). The serum AFP level was tested using a radio-immunoassay kit (Serono Diagnostic SA, Coinsin/VD, Switzerland).

### Statistical Analysis

The primary end point was overall survival—this was calculated from the diagnosis of HCC to death, the last patient visit, or loss to follow-up. The baseline characteristics and outcomes were selected according to the European Association for the Study of the Liver guidelines from 2001.^31^ Fisher exact test or a Chi-squared test with Yates’ correction was used to compare categorical variables when appropriate, and the Mann–Whitney *U* test was used to compare continuous variables. The cumulative overall survival rates and recurrent rates after curative therapies were estimated using the Kaplan–Meier method and compared using Cox proportional hazards model.

Variables with statistical significance (*P* < 0.05) or approximate significance (*P* < 0.1) by univariate analysis were subjected to multivariate analysis using a forward stepwise logistic regression model. A 2-tailed values of *P* < 0.05 was considered statistically significant. All statistical analyses were performed using SPSS 17.0 for Windows (SPSS. Inc., Chicago, IL).

## RESULTS

### Baseline Clinical Characteristics

The baseline demographic data are shown in Table 1 and Supplementary Table S1–S4, http://links.lww.com/MD/A488. Patients in the BCLC stage 0 group were significantly younger than those in the other groups (*P* = 0.001). Furthermore, patients with chronic HCV infection were more common in the BCLC A2–A4 group than those in stage 0–A1 group.

**TABLE 1:**
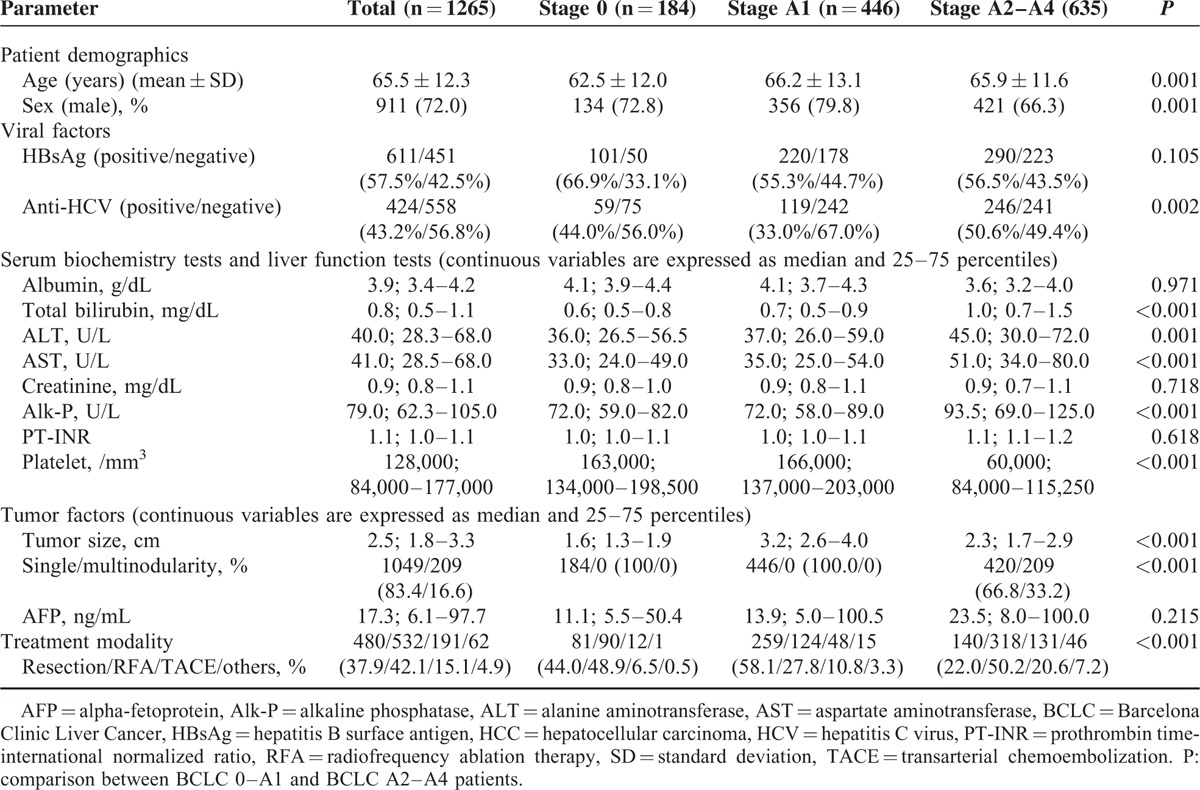
Demographic Data of Early Stage HCC Patients

Liver functional reserve including total bilirubin was relatively poor for patients in the BCLC A2–A4 group (*P* < 0.001). They also had lower platelet counts, and higher levels of serum alanine aminotransferase, aspartate aminotransferase, and alkaline phosphatase (*P* < 0.001) versus other groups of patients.

The BCLC A1 group had larger tumors than the BCLC 0 or BCLC A2–A4 group. The serum AFP levels are comparable to those in other patient groups (*P* = 0.215). The rate of patients who underwent curative therapy was higher in the BCLC 0–A1 group versus stage A2–A4 (88.0% vs 72.2%, *P* < 0.001).

### Overall Survival of Patients in the BCLC Stage 0 and A HCC

**FIGURE 2:**
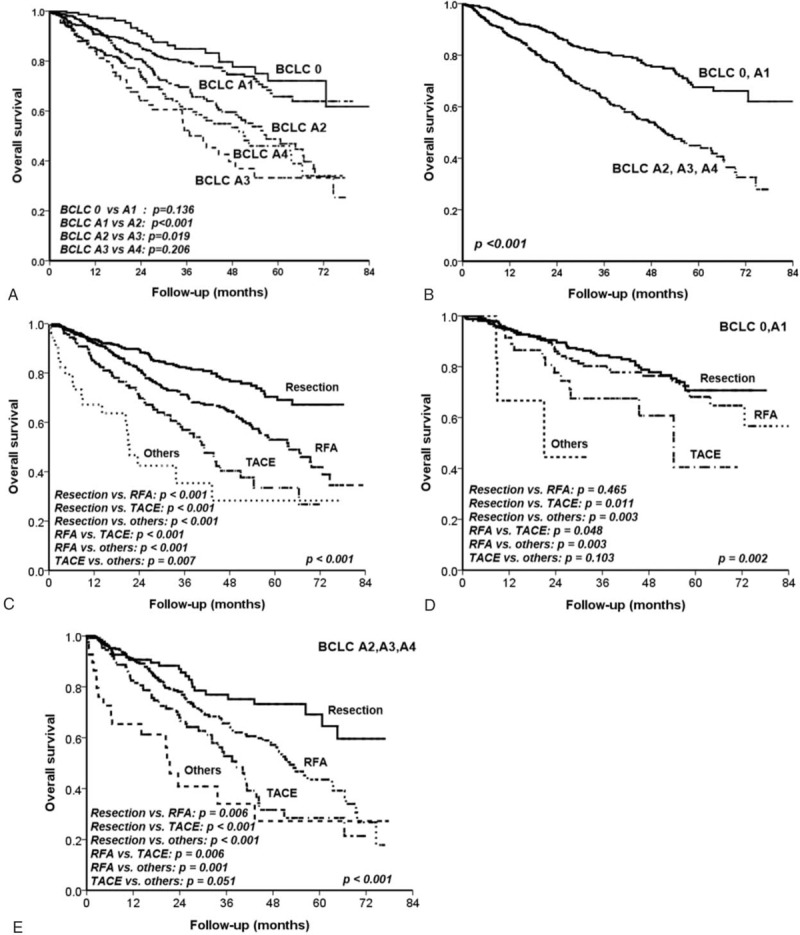
Comparison of cumulative overall survival rates stratified by BCLC substage and treatment modality. (A) Patients in the stage 0 or A1 group had a significantly higher overall survival rate than those in the other substages (all *P* < 0.001). BCLC 0 versus A1 (*P* = 0.136) had comparable overall survival rates. (B) The overall survival rates were higher in the BCLC stage 0–A1 group than that in the BCLC stage A2–A4 group (*P* < 0.001). (C) Patients who underwent resection surgery had the highest overall survival rates than the other groups (all *P* < 0.001). (D) For patients in BCLC stage 0–A1 group, RFA and resection had comparable overall survival rates (*P* = 0.465); both of them were significantly better than other treatment modalities. (E) For patients in BCLC stage A2–A4, patients who underwent resection had the highest overall survival rates than other treatment modalities including RFA. BCLC = Barcelona Clinic Liver Cancer, RFA = radiofrequency ablation therapy.

After a median follow-up of 21.0 (25–75 percentiles 8.9–42.1) months, 305 patients died and 960 were still alive on their last visit. When stratified by BCLC substage, the cumulative overall survival rates at 3 and 5 years were 84.9% and 72.1% in the BCLC stage 0 group; 79.5% and 65.8% in the stage A1 group; 69.6% and 48.8% in the A2 group; 52.2% and 33.2% in the A3 group; and 60.8% and 46.0% in the BCLC A4 group, respectively. As shown in Figure 2A and B, patients in the stage 0–A1 group had a significantly higher overall survival rate than those in the other substages (all *P* < 0.001). The BCLC A2 group had a significantly higher overall survival rate than the BCLC A3 group (*P* = 0.019). The BCLC 0 versus A1 (*P* = 0.136), A2 versus A4 (*P* = 0.142), and BCLC A3 versus A4 (*P* = 0.206) had comparable overall survival rates.

Stratified by treatment modalities, patients who underwent resection surgery had the highest overall survival rate versus other therapies followed by RFA, TACE, and other therapies (Fig. 2C). All of the treatment modalities showed significantly different overall survival statistics.

### Multivariate Analysis of Independent Risk Factors Associated With Poor Prognosis

As the substages of BCLC stage A are classified by PHT and jaundice, we applied 2 multivariate analysis models to minimize the potential confounding effects of these parameters. In model I, the BCLC stage was enrolled, but platelet count and bilirubin levels were not entered into the multivariate analysis. In model II, we selected platelets and bilirubin, but not BCLC stage for multivariate analysis

In model I, serum albumin levels ≤3.5 g/dL (*P* = 0.009), AFP >20 ng/mL (*P* < 0.001), tumor size >3 cm (*P* = 0.003), BCLC stage A2–A4 (*P* = 0.013), and treatment modality (resection as reference, RFA, hazard ratio, HR 1.598, 95% confidence interval CI: 1.142–2.237, and *P* = 0.006; TACE, HR 2.224, 95% CI: 1.507–3.282, and *P* < 0.001; and others, HR 3.707, 95% CI: 2.076–6.620, and *P* < 0.001) were the independent risk factors associated with poor overall survival (Table 2).

**TABLE 2:**
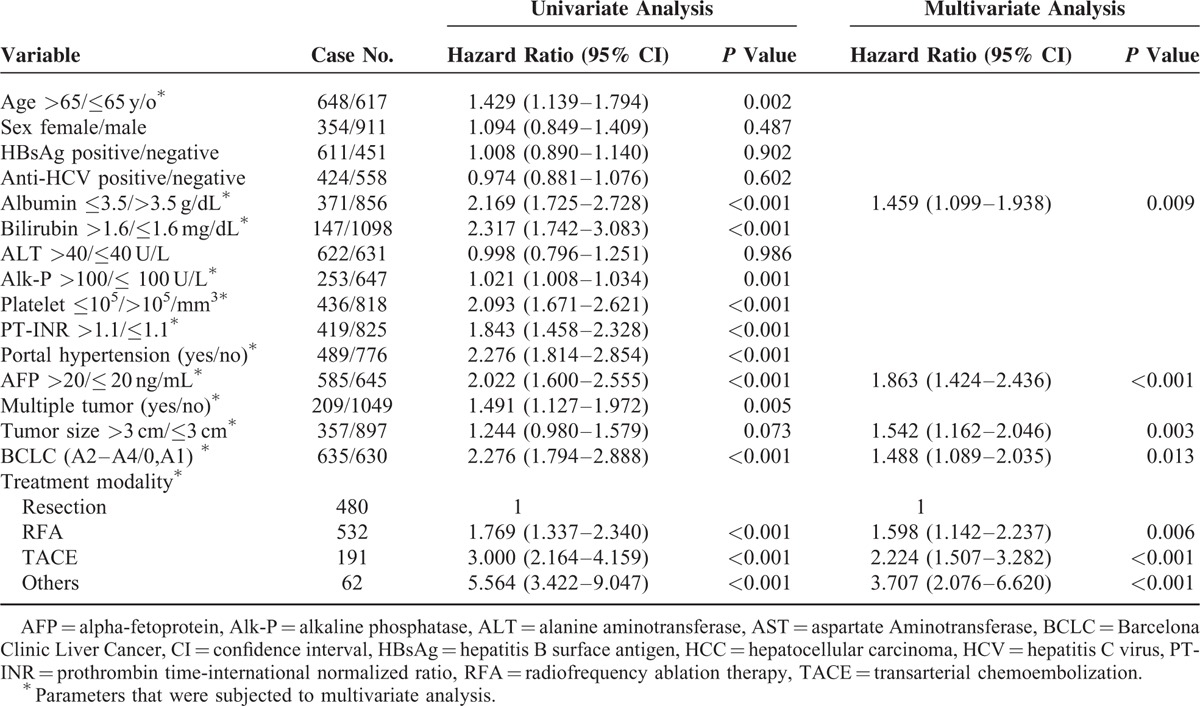
Factors Associated With Poor Overall Survival in BCLC Stage 0 and A HCC Patients in Model I

In model II, serum bilirubin levels >1.6 g/dL (HR 1.540, 95% CI: 1.080–2.196, and *P* = 0.017), platelet ≤10^5^/mm^3^ (HR 1.362, 95% CI: 1.012–1.833, and *P* = 0.042), AFP >20 ng/mL (HR 1.949, 95% CI: 1.491–2.549, and *P* < 0.001), tumor size >3 cm (HR 1.508, 95% CI: 1.143–1.989, and *P* = 0.004), and treatment modality (RFA, HR 1.709, 95% CI: 1.223–2.389, and *P* = 0.002; TACE, HR 2.391, 95% CI: 1.619–3.530, *P* < 0.001; and others, HR 4.138, 95% CI: 2.329–7.352, and *P* < 0.001) were the independent risk factors associated with poor overall survival.

### Prognoses of Patients in the BCLC Stage 0–A1 HCC

The prognoses were significantly better in patients who had tumors in stage 0–A1, and we further assessed these patients for prognostic factors. Of the 630 patients in BCLC stage 0–A1, 340 underwent resection, 214 patients received RFA, 60 patients underwent TACE, and the remaining 16 patients received other therapies.

As shown in Table 3 and Figure 2D, multivariate analysis showed that serum albumin ≤3.5 g/dL (*P* = 0.005), AFP >20 ng/mL (*P* = 0.012), tumor size >3 cm (*P* = 0.028), and treatment modalities (resection as reference, RFA, *P* = 0.109; TACE, and *P* = 0.035; and other therapies, *P* = 0.012) were the independent risk factors predicting poor prognoses in BCLC stage 0 or A1 patients.

**TABLE 3:**
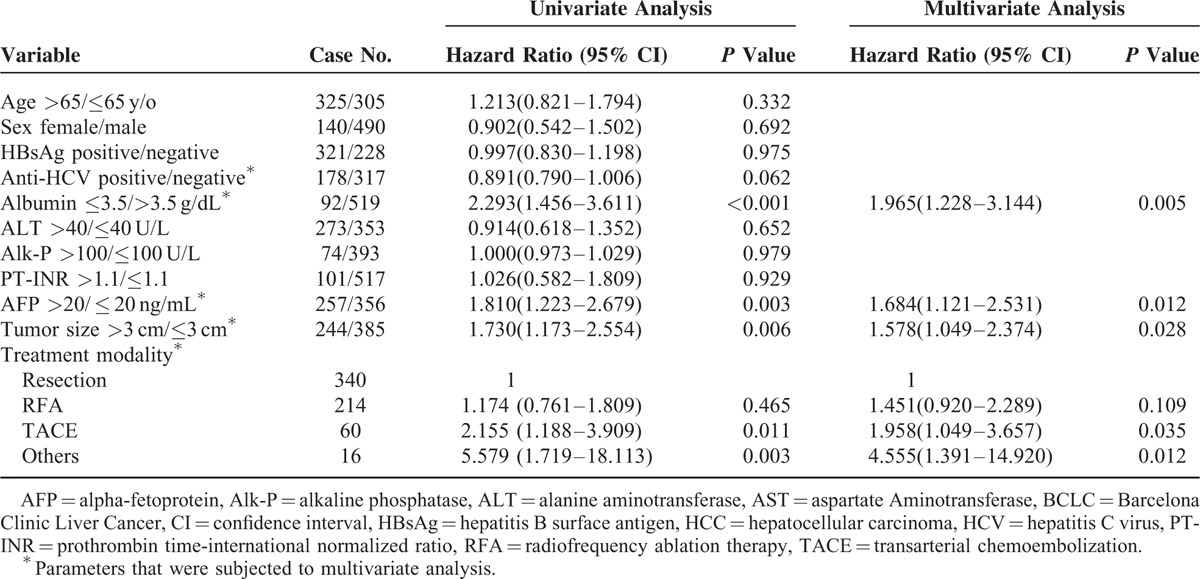
Factors Associated With Poor Overall Survival in BCLC Stage 0 or A1 HCC Patients

### Prognoses of Patients in the BCLC Stage A2–A4 HCC

Of the 635 patients in BCLC stage A2–A4, the number of patients undergoing resection, RFA, TACE, and other therapies were 140, 318, 131, and 46, respectively. Multivariate analysis showed that AFP >20 ng/mL (*P* < 0.001), tumor size >3 cm (*P* = 0.022), and treatment modalities (resection as reference, RFA, *P* = 0.012; TACE, *P* = 0.001; and other therapies, *P* < 0.001) were the independent poor prognostic factors in these patients (Table 4, Fig. 2E).

**TABLE 4:**
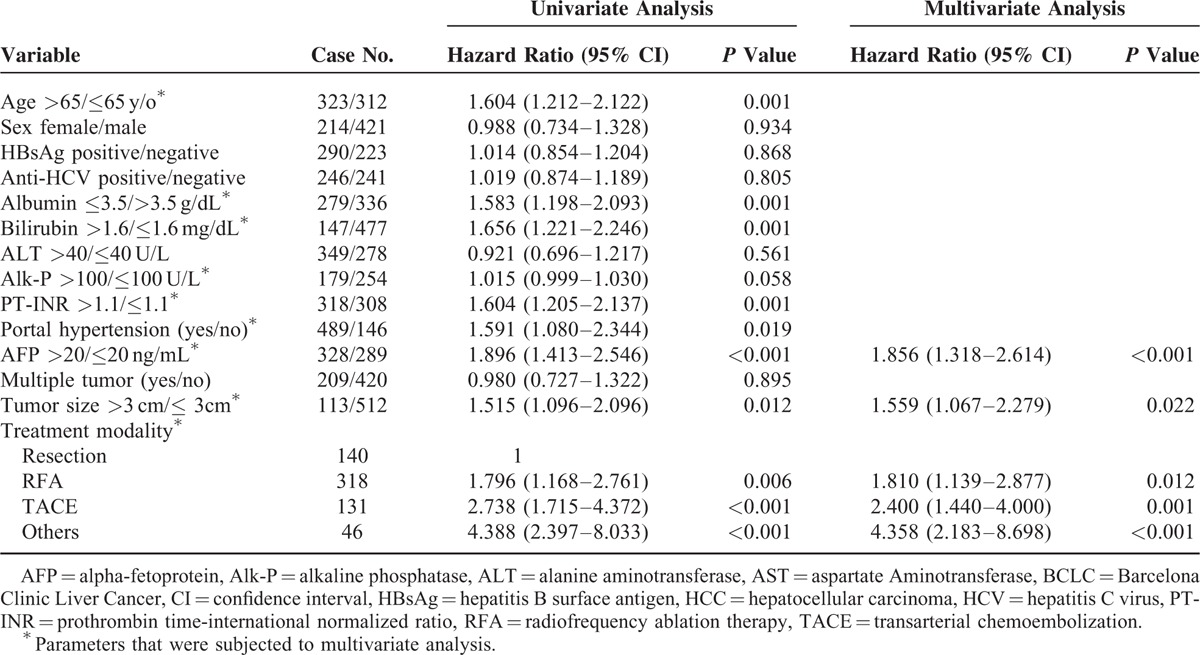
Factors Associated With Poor Overall Survival in BCLC Stage A2–A4 HCC Patients

### Comparison of Prognosis Among Different BCLC Substages Stratified by Treatment Modalities

**FIGURE 3:**
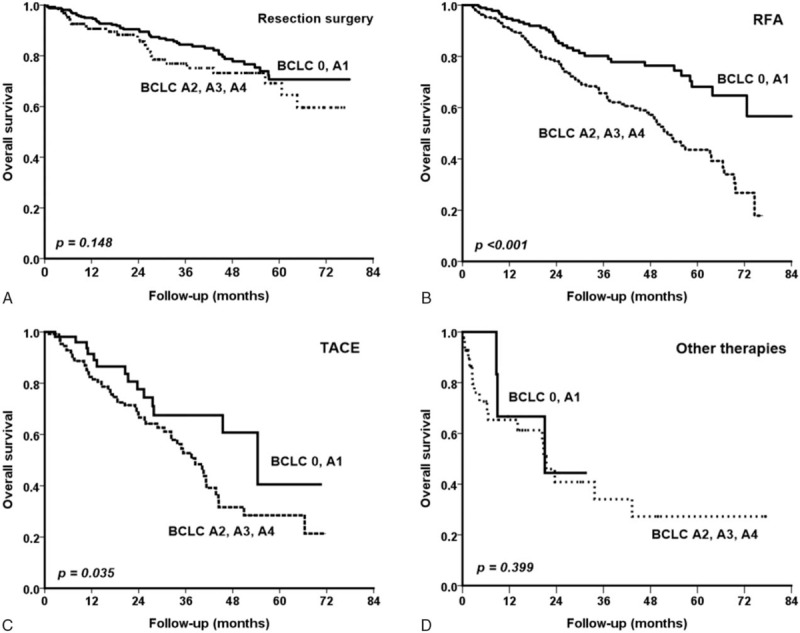
Comparison of overall survival rates among different BCLC substages with the same treatment modality. (A) In the resection surgery group, patients in the stage 0–A1 group had a comparable overall survival rate with those in the stage A2–A4 (*P* = 0.148). (B) In the RFA group, patients in the stage 0–A1 group had a significantly higher overall survival rate than those in the stage A2–A4 (*P* < 0.001). (C) In the TACE group, patients in the stage 0–A1 group had a higher overall survival rate than those in the stage A2–A4 (*P* = 0.035). (D) For those underwent other therapy modalities, patients in the stage 0–A1 group had a comparable overall survival rate with their counterpart (*P* = 0.399). BCLC = Barcelona Clinic Liver Cancer, RFA = radiofrequency ablation therapy, TACE = transarterial chemoembolization.

Subsequently, we assessed the effect of the same treatment modality in determining the prognosis of patients among different BCLC substages. For those who underwent resection surgery, patients in the stage 0–A1 group had a comparable overall survival rate with those in the stage A2–A4 (Fig. 3A, *P* = 0.148). In the RFA group and TACE group, patients in the stage 0–A1 group had a significantly higher overall survival rate than those in the stage A2–A4 (Fig. 3B, *P* < 0.001 and Fig. 3C, *P* = 0.035, respectively). For those who underwent other therapy modalities, patients in the stage 0–A1 group had a comparable overall survival rate with their counterpart (Fig. 3D; *P* = 0.399).

### Comparison of Resection Surgery and RFA Stratified by BCLC Substages

**FIGURE 4:**
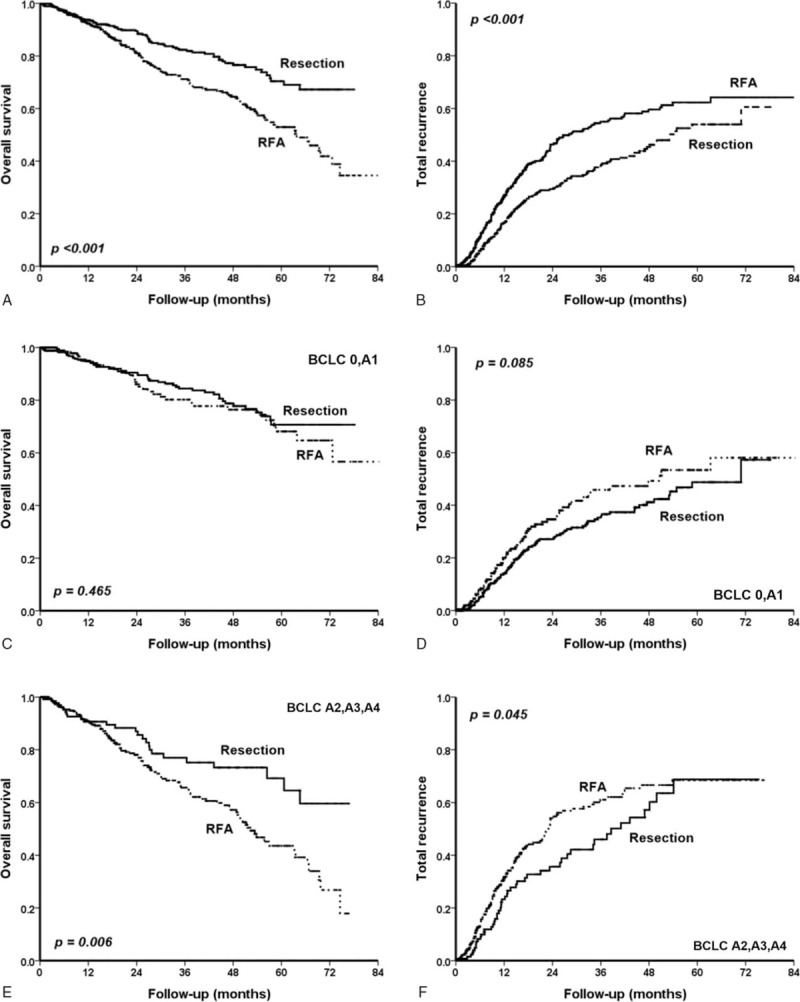
Comparison of overall survival and recurrence rates between RFA and resection surgery for HCC stratified by BCLC substage. In a total cohort, patients who underwent resection had a higher overall survival rate (A, *P* < 0.001) and lower rate of recurrence (B, *P* < 0.001) versus those receiving RFA. For BCLC stage 0–A1 HCC patients, there was no statistical significance between RFA and resection in overall survival (C, *P* = 0.465); resection did have a trend of lower incidence of recurrence versus RFA (D, *P* = 0.085). Among the BCLC stage A2–A4 patients, the resection group had a better prognoses than the RFA group both in term of overall survival (E, *P* = 0.006) and recurrence (F, *P* = 0.045). BCLC = Barcelona Clinic Liver Cancer, HCC = hepatocellular carcinoma, RFA = radiofrequency ablation therapy.

We next compared the treatment efficacy between resection surgery and RFA in these patients. The cumulative overall survival rates at 3 and 5 years were 82.4% and 70.4% in the resection group and 71.2% and 52.9% in the RFA group, respectively (Fig. 4A, *P* < 0.001). Moreover, the cumulative recurrence rates at 3 and 5 years were 39.2% and 53.9% in the resection surgery group; and 55.0% and 62.3% in the RFA group, respectively (Fig. 4B, *P* < 0.001).

In the BCLC stage 0–A1 group, the cumulative overall survival rates at 3 and 5 years were 84.4% and 70.7% in the resection surgery group and 80.2% and 68.1% in the RFA group, respectively (Fig. 4C, *P* = 0.465). Moreover, the cumulative recurrence rates at 3 and 5 years were 35.3% and 48.7% in the resection surgery group; and 45.8% and 53.4% in the RFA group, respectively (Fig. 4D, *P* = 0.085). This suggested that although they had similar overall survival rates, patients who underwent resection surgery had a trend of lower incidence of recurring than those treated by RFA in patients with BCLC stage 0 or A1.

For patients in BCLC stage A2–A4, the cumulative overall survival rates at 3 and 5 years were 77.0% and 69.2% in the resection surgery group, 65.6% and 43.6% in the RFA group, respectively (Fig. 4E, *P* = 0.006). Moreover, the cumulative recurrence rates at 3 and 5 years were 46.0% and 68.7% in the resection surgery group, 61.1% and 68.5% in the RFA group, respectively (Fig. 4F, *P* = 0.045). Patients who underwent resection surgery had better prognoses than those receiving RFA both in terms of overall survival and recurrence.

## DISCUSSION

The BCLC staging system is a prognostic system and offers guidance for treatment decisions based on tumor burden, liver functional reserve, and performance status.^1,2^ There are several major findings in this study. First, we demonstrated that the substages of early-stage HCC provided a good prediction for prognosis. Second, patients in BCLC stage 0–A1 had higher chances of undergoing curative therapy. They also had similar prognoses and were significantly better than those in the stages A2–A4. Third, patients who underwent surgical resection had a higher overall survival rate than those receiving other treatment modalities. This result suggests that curative therapies, especially hepatic resection, should be performed in early-stage HCC.^32,33^

In this study, patients in BCLC stages 0 and A1 had comparable prognoses (*P* = 0.136). Both of them had significantly higher overall survival rates than those in stages A2–A4 (all *P* < 0.001). Furthermore, patients in BCLC stages A2 and A4 had similar overall survival rates (*P* = 0.142), BCLC stage A3 had the worst outcome due to both increased bilirubin and clinically significantly elevated PHT. This implied that BCLC stage A patients are not all the same. This heterogeneity may have an impact on survival.

In our cohort, the cumulative survival rate 5 years after resection was 70.7% for the best candidates who had single HCC without PHT and with normal serum bilirubin levels. This was consistent with the findings of the BCLC staging system proposed by Professor Bruix and Llovet in 1999. They reported that the cumulative 5-year survival rate after resection surgery was 74%.^26^ We also demonstrate that serum albumin and AFP levels, tumor size, BCLC substage, and treatment modalities are strong predictors of outcome. This is consistent with the results from previous studies including both tumor factors (tumor size, number, vascular invasion, tumor cell differentiation, etc.) and field factors (grade of hepatic inflammation, stage of fibrosis, PHT, liver functional reserve, viral replication, etc.) that have been shown to influence the prognosis of HCC patients.^18,34–36^

Assessment of liver functional reserve is critical for the management of HCC because cirrhosis is a competing cause of death. For patients with early-stage HCC, field factors play more important roles than advanced tumor stages. The substages of early-stage HCC are classified by PHT, jaundice, and number of tumors.^26^ This stratification arises from studies on the BCLC groups that reported that PHT and increased serum bilirubin levels are independent predictors of an elevated risk of hepatic decompensation and mortality after surgical resection in patients with compensated cirrhosis.^20^

PHT is a hepatic-vein pressure gradient more than 5 mmHg. Clinically significant PHT is indicated when the hepatic-vein pressure gradient is above 10 mmHg—this is also the threshold for development of gastroesophageal varices.^37^ A large study of Italian patients with HCC showed that the prevalence of EV in patients with HCC is around 60% to 80%.^38^ Of note, HCC patients with EV are associated with a significantly poorer prognosis than those without EV.

Serum albumin and bilirubin levels are the 2 most significant independent prognostic factors to predict hepatic events in patients with cirrhosis.^37^ A new prognostic score, the albumin–bilirubin (ALBI) score, which incorporates serum albumin and bilirubin concentrations, can offer an evidence-based, objective, and discriminatory method of assessing liver function with good prognostic performance in HCC.^39^ In our cohort, when assessing the independent risk factors predicting poor overall survival by multivariate analysis, model I demonstrated that BCLC substage is correlated to poor prognosis. In model II, when the BCLC substage is not enrolled, its 2 major determinants (platelet count and serum bilirubin level) could predict outcomes. This validated the critical role of the BCLC substages in determining the prognoses of Asian patients with early-stage HCC.

Curative therapies including surgical resection, local ablation therapies, and liver transplantation are recommended as first-line treatment modalities in early-stage HCC patients.^1,2^ Of the curative therapies, liver transplantation is limited by organ donor shortage. Resection surgery and RFA are the major therapies in daily Taiwanese practice.^24^ Treatment modalities are related to long-term outcomes and are an independent predictor of HCC patient prognosis.^14,27^ However, underutilization of curative treatments for early-stage HCC is not uncommon due to tumor factors, performance status, and availability of treatment modalities.^32,40,41^

In this study, 80.0% of the early-stage HCC patients received curative treatment, but this is still not good enough. To better match the treatment modality with the BCLC staging system guidelines in the real world, a multidisciplinary HCC team approach may help physicians and encourage patients to choose the optimal curative treatment modalities.

The efficacy of RFA with surgical resection in the treatment of early-stage HCC is still actively debated.^24,42,43^ In this study, surgical resection provides better prognoses both in long-term overall survival and in recurrence verus RFA in patients with early-stage HCC. The superiority of resection was more apparent in BCLC stage A2–A4, which considered PHT or tumor number. Moreover, the overall survival rates were comparable between BCLC stage 0–A1 and A2–A4 in patients who underwent resection surgery (Fig. 3). Consequently, our study also confirms that neither PHT nor multinodular tumors are contra-indicated for resection surgery.^44–46^ This provides a more favorable outcome than other treatment modalities and is therefore recommended for such patients if there is no contraindication for operation.

The role of viral factors in HCC prognosis remains controversial.^4,35,47–49^ In this study, patients with chronic HCV infection had a higher recurrence rate than their counterparts. Previous studies showed that in early-stage HCC, patients with HBV infection had better prognosis than those with HCV due to better liver reserve and less hepatic fibrosis.^49^ Furthermore, our previous study demonstrated that HCV-related HCC correlated with overexpression of twist, a major regulator of epithelial-mesenchymal transition that is critical for the induction of invasiveness and metastasis for human cancers.^50^

There are some limitations to this study. First, our study included HCC patients from a single tertiary center. Second, interobserver bias may exist in the amount of ascites and the degree of hepatic encephalopathy for Child–Pugh scoring—this is an important parameter of BCLC staging. Third, our cohort did not include any HCC patients who underwent liver transplantation as an initial treatment modality. Thus, this data may not be relevant to medical centers that perform many liver transplants. Fourth, we could not assess the effect of the same treatment modality in determining the prognosis of HCC patients in each BCLC substage due to relatively small number of patients in some substages. Further large-scale prospective studies are needed to elucidate this issue.

## CONCLUSIONS

The substages of the BCLC staging system based on PHT, serum bilirubin levels, and tumor numbers was useful in predicting survival in patients with early-stage HCC. Patients with single tumor larger than 2 cm but without significant PHT or jaundice had similar prognosis to those in BCLC stage 0. Moreover, curative therapies, especially hepatic resection, were crucial in determining the prognosis and should be performed in early-stage HCC.
